# Validation of blood vitamin A concentrations in cattle: comparison of a new cow-side test (iCheck™ FLUORO) with high-performance liquid chromatography (HPLC)

**DOI:** 10.1186/s12917-017-1042-3

**Published:** 2017-05-10

**Authors:** Jens Raila, Chiho Kawashima, Helga Sauerwein, Nadine Hülsmann, Christoph Knorr, Akio Myamoto, Florian J. Schweigert

**Affiliations:** 1 Institute of Nutritional Science, University of Potsdam, Arthur-Scheunert-Allee 114-116, 14558 Nuthetal, Germany; 2 Obihiro University of Agriculture and Veterinary Medicine, Obihiro, Hokkaido 080-8555 Japan; 3 Institute for Animal Science, Physiology and Hygiene, University of Bonn, Katzenburgweg 7-9, 53115 Bonn, Germany; 4 Department of Animal Sciences, Biotechnology and Reproduction of Livestock, Georg-August-University Goettingen, Burckhardtweg 2, 37077 Goettingen, Germany

**Keywords:** Cattle, Vitamin A, Biomarker, Blood, Method comparison, Cow-side assay

## Abstract

**Background:**

Plasma concentration of retinol is an accepted indicator to assess the vitamin A (retinol) status in cattle. However, the determination of vitamin A requires a time consuming multi-step procedure, which needs specific equipment to perform extraction, centrifugation or saponification prior to high-performance liquid chromatography (HPLC).

**Methods:**

The concentrations of retinol in whole blood (*n* = 10), plasma (*n* = 132) and serum (*n* = 61) were measured by a new rapid cow-side test (iCheck™ FLUORO) and compared with those by HPLC in two independent laboratories in Germany (DE) and Japan (JP).

**Results:**

Retinol concentrations in plasma ranged from 0.033 to 0.532 mg/L, and in serum from 0.043 to 0.360 mg/L (HPLC method). No significant differences in retinol levels were observed between the new rapid cow-side test and HPLC performed in different laboratories (HPLC vs. iCheck™ FLUORO: 0.320 ± 0.047 mg/L vs. 0.333 ± 0.044 mg/L, and 0.240 ± 0.096 mg/L vs. 0.241 ± 0.069 mg/L, lab DE and lab JP, respectively). A similar comparability was observed when whole blood was used (HPLC vs. iCheck™ FLUORO: 0.353 ± 0.084 mg/L vs. 0.341 ± 0.064 mg/L). Results showed a good agreement between both methods based on correlation coefficients of r^2^ = 0.87 (*P* < 0.001) and Bland-Altman blots revealed no significant bias for all comparison.

**Conclusions:**

With the new rapid cow-side test (iCheck™ FLUORO) retinol concentrations in cattle can be reliably assessed within a few minutes and directly in the barn using even whole blood without the necessity of prior centrifugation. The ease of the application of the new rapid cow-side test and its portability can improve the diagnostic of vitamin A status and will help to control vitamin A supplementation in specific vitamin A feeding regimes such as used to optimize health status in calves or meat marbling in Japanese Black cattle.

**Electronic supplementary material:**

The online version of this article (doi:10.1186/s12917-017-1042-3) contains supplementary material, which is available to authorized users.

## Background

Vitamin A (retinol) is an essential micronutrient not only to ascertain vision but also to modulate growth and development [1–4]. Cattle as well as other herbivorous animals are unable to synthesize retinol de novo and they have to obtain their vitamin A primarily from dietary *β*-carotene, which is converted into retinol within the enterocytes [5]. Independently from provitamin A activity, *β*-carotene can be absorbed intact in the gut and is considered to have positive effects on reproductive performance and immune response in cattle [6]. All green forages are rich in *β*-carotene and thus provide a high vitamin A value mainly during pasture conditions [7]. Low concentrations of vitamin A in blood resulting from dietary *β*-carotene restriction or lacking in vitamin A supplementation and are related to an increased risk of disease [8]. Neonate calves are particularly prone to develop symptoms of vitamin A deficiency, because they have low blood vitamin A concentrations and insufficient hepatic vitamin A stores at birth [9]. Low hepatic vitamin A storage is associated with stillborn and postnatal calf losses as a consequence of an insufficient vitamin A intake of the mother cows especially during non-grazing conditions [10]. In addition, cases of dermatopathy associated with hypovitaminosis A were recently described in juvenile Angus calves, which could be successfully treated with parenteral vitamin A supplementation [11]. Thus, the measurement of blood retinol concentrations in mother cows as well as in neonate and juvenile calves is a prerequisite for evaluation of their health status.

Low vitamin A plasma concentrations are generally found in Japanese Black cattle and are associated with an increased intramuscular or marbling adipose deposition without influencing other adipose depots [12–15]. Thus, to optimize the desired intramuscular fat marbling that increases the carcass value, Japanese Black fattening cattle are provided with a high concentrate diet deficient in vitamin A or *β*-carotene. It was suggested that optimal marbling scores are obtained when animals are kept very close to health deteriorating low vitamin A plasma concentrations in a range of 100–150 μg/L (40–50 IU/dl) over a period of usually 12 month during the fattening period [16]. However, in Japanese Black cattle low vitamin A plasma concentrations have been reported to be associated with an impaired immune function [17] and increased morbidity and mortality [18]. Therefore, blood vitamin A levels in Japanese Black cattle needs to be carefully monitored and nutritionally controlled. Additional reduced vitamin A plasma concentrations are also observed as a consequence of the acute phase reaction e.g. during inflammation or around parturition [19–21]. Thus, due to the restrict feeding management, the induced vitamin A deficiency in Japanese Black cattle can cause a serious health problem if not monitored and nutritionally controlled properly.

Under practical management conditions, vitamin A deficiency is difficult to diagnose and marginal deficiency may exist without obvious deficiency symptoms. Measurement of retinol in blood plasma is an appropriate marker to assess vitamin A status but it is currently a time consuming and cost-intensive multi-step procedure. Specific equipment is needed to perform extraction and centrifugation or saponification. Vitamin A is finally measured by colorimetric methods [22] or high performance liquid chromatography (HPLC) [23]. All preanalytic steps and the final analyses have to be performed in qualified analytical laboratories by highly trained personal. Alternatively suggested methods such as thin layer chromatography are similar complicated and not very reliable [24]. So far, however, no method has been described which is also able to measure vitamin A directly at cow-side in the barn or even determining vitamin A directly from whole blood prior to centrifugation.

This study presents data for a new, fast, easy to perform and laboratory-independent test to measure retinol in whole blood, plasma or serum. Results are compared with those obtained by HPLC as the reference method at two independent laboratories. The new assay is based on a separation and extraction principal published for the determination of *β*-carotene in blood plasma [25] as well as human and cow milk [26, 27].

## Methods

The comparison was performed at two independent laboratory sites at the University of Potsdam, Germany (lab DE) and at the Obihiro University, Japan (lab JP).

### Animals and sampling

#### Lab DE

A total of 132 blood samples were collected from dairy cows (*n* = 40) and bulls (*n* = 92) into EDTA-evacuated tubes (Saarstedt, Nümbrecht, Germany) and immediately separated by centrifugation (1500 × *g*; 10 min; 4 °C). The plasma was frozen at −80 °C and analyzed for their vitamin A concentrations with both methods (HPLC and iCheck™ FLUORO) within three months. In addition, 10 blood samples were taken from dairy cattle and collected into EDTA-evacuated tubes (Saarstedt) and analyzed freshly for their vitamin A content within 24 h after acquisition both in whole blood and in plasma after removal of the erythrocytes by centrifugation (1500 × *g*; 10 min; 4 °C). All samples were obtained from institutional farms (Research Station Frankenforst, Faculty of Agriculture, University of Bonn, Koenigswinter, Germany and Verein Ostfriesischer Stammviehzuechter, Leer, Germany).

#### Lab JP

A total of 61 blood samples from Holstein dairy cows (*n* = 29) and Japanese Black cattle (*n* = 32) were obtained by caudal venipuncture using non-heparinized and silicone-coated 9-mL tubes (Venoject, Autosep, Gel and Clot. Act., VP-AS109K; Terumo Corporation, Japan). Samples were collected from the institutional farm of the Obihiro University of Agriculture and Veterinary Medicine, Japan and Akiyama Farm Animal Clinic, Japan. To obtain serum, blood samples were coagulated for 15 min at 38 °C in an incubator. All tubes were centrifuged at 2000 × *g* for 20 min at 4 °C, and serum samples were kept at −30 °C until analysis within three months.

### Vitamin A determination by HPLC

#### HPLC lab DE

A modified gradient reverse-phase HPLC system (Shimadzu, Duisburg, Germany) was used [28]. Briefly, vitamin A as retinol or retinyl esters was extracted from plasma and thereafter separated on a reverse-phase column (ReproSil 70 C18 column, 200 × 3.0 mm; inside diameter 5 μm; Dr. Maisch GmbH, Ammerbuch, Germany). The solvent system consisted of solvent A with methanol and solvent B with ethyl acetate at a flow rate of 0.5 mL/min (pump LC 20-AD, Shimadzu). Retinol was identified based on retention time in comparison to an external standard (Sigma, Munich, Germany) by use of a photodiode array detector (PDA SPDM-20A, Shimadzu). Vitamin A was quantified by measuring the absorption at 325 nm.

#### HPLC lab JP

The concentrations of retinol in serum were determined by HPLC, as described previously [29]. Extraction efficiency was 96%. Intra- and inter-assay CVs averaged 2.1% and 3.3%, respectively.

### Vitamin A determination by a portable fluorometer

The novel cow-side test for vitamin A consists of an extraction unit that contains all necessary chemicals for extraction and separation and a portable fluorometer (iCheck™ FLUORO, BioAnalyt GmbH, Teltow, Germany). The combination of these two components enables to extract vitamin A from whole blood without prior separation of plasma in a single step at cow-side. The analysis of whole blood, blood plasma or serum with the new and innovative assay system was done as recommended by the manufacturer. A total volume of 500 μl of whole blood, plasma or serum was injected with an included syringe into the extraction and measuring vials. Thereafter it was shaken intensively for 10 s and then let settle for 5 min until complete phase separation. Finally, the vials were inserted into the fluorometer and measured. The quantification of vitamin A in whole blood, plasma or serum is based on the specific autofluorescent characteristics of excitation and emission wavelengths. Since that autofluorescence is based on the retinol moiety, any form of vitamin A is included in the quantification. Results were recorded as μg/L whole blood, plasma or serum.

### Statistical analyses

The data were analysed using SPSS version 23.0 software (SPSS, Munich, Germany). The results obtained by HPLC and the new fluorometric method were judged for method acceptability as suggested for clinical laboratories [30]. Associations between the results were determined with Pearson correlation method. Mean results obtained by HPLC and fluorometer were compared with a paired *t* test. The difference of results obtained from HPLC and fluorometer were analyzed by one sample *t* test. A Bland-Altman bias plot [31] was done to determine the analytical accuracy of the two analytical methods. The analytical detection limit was defined as proposed in the concept of ‘functional sensitivity’. This is defined as the lowest concentration of an assay that can be measured with an inter-assay CV of 20% [32]. Values of *P* < 0.05 were assigned as statistically significant.

## Results and discussion

The concentrations of retinol in plasma or serum as determined by HPLC and the new method ranged from 0.033 mg/L to 0.532 mg/L for plasma in Lab DE and from 0.043 mg/L to 0.360 mg/L for serum in Lab JP (Additional file 1: Table S1). No significant differences were observed between both methods at two different laboratories for the average values obtained (HPLC vs. new method: 0.320 ± 0.047 mg/L vs. 0.333 ± 0.044. mg/L; mean ± SD in Lab DE and 0.240 ± 0.096 mg/L vs. 0.241 ± 0.069 mg/L; mean ± SD in Lab JP).

The analytical advantage of the new innovative test method is to measure retinol from whole blood directly. This avoids the time consuming and limiting preanalytical step of centrifugation to obtain plasma or serum. In consequence, the new assay can be directly used in the barn. When the subset of 10 whole blood samples were assayed for retinol by the new test method and compared to plasma levels assessed by HPLC in Lab DE, a similar good agreement was found as for the use of plasma (HPLC plasma: 0.353 ± 0.084 mg/L vs. new test plasma: 0.340 ± 0.063 mg/L vs. new test whole blood 0.341 ± 0.064 mg/L; mean ± SD; see Additional file 2: Table S2). There were significantly correlations between concentrations of retinol measured in whole blood by the new method and retinol measured in plasma by HPLC (r^2^ = 0.84; *P* < 0.001) as well as retinol measured in plasma by the new method (r^2^ = 0.87; *P* < 0.001). The average packed cell volume of the 10 whole blood samples in this study was 31.7 ± 2.7% (mean ± SD).

**Fig. 1:**
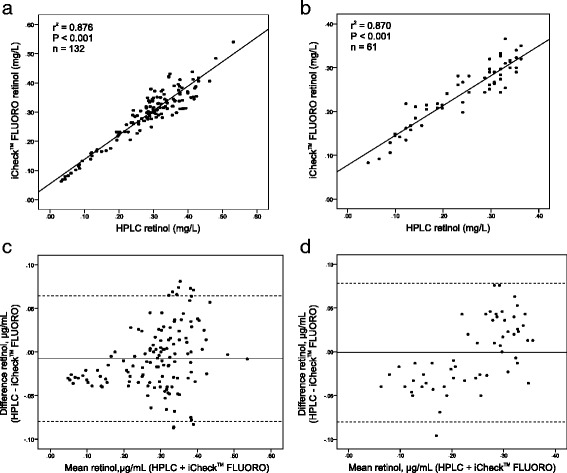
Correlation analyses demonstrate close association between plasma retinol as measured by HPLC and iCheck™ fluorometer in lab Germany (**a**) and lab Japan (**b**). Bland-Altman plot showing the mean difference (solid line) and the 95% confidence interval (dotted lines) of the retinol values in blood plasma obtained by HPLC and iCheck™ fluorometer in lab Germany (**c**) and lab Japan (**d**)

The comparison between plasma or serum samples assayed with the two different methods in Lab DE (plasma) and Lab JP (serum) indicated a strong correlation between methods (Fig. 1a, b). Results show that the novel cow-side test for vitamin A correlated very well with HPLC analysis (r^2^ = 0.876 and 0.870, Lab DE and Lab JP, respectively, both *P* < 0.001). The calculated differences in retinol concentrations obtained by HPLC and fluorometer were not significantly different (*P*-values >0.05 for both Lab DE and Lab JP) indicating an acceptable level of agreement between the two assay methods. Based on the Bland-Altman plot (Fig. 1c, d) no systematic error and a good level of agreement occurred between the two methods and only 4% of the differences in measured values felt outside the 95% acceptability limits.

Based on multiple determinations of selected samples the intra-assay and inter-assay precision were calculated for each method (Lab DE). In average, the coefficient of variance (CV) was in an order of magnitude generally accepted for average analysis of this kind. The CV was slightly higher in the HPLC method compared to the new method (5.3% vs. 2.3%, respectively).

## Conclusions

The comparison of the new cow-side test (iCheck™ FLUORO) to measure vitamin A in cattle whole blood, blood plasma or serum showed a very good agreement, reliability and accuracy with the cumbersome, time-intensive and expensive HPLC analytical technique. The assay allows the determination of vitamin A directly in whole blood within 5 min even at cow-side at the barn without a further sample preparation. Because all chemicals are sealed in the vial, the staff is not directly exposed to potentially hazardous organic chemicals. Additionally, the limited volume of organic solvent necessary is positive with regard to environmentally and ecologically critical waste. The novel test realizes important aspects of a cow-side assay: it is sensitive, specific and rapid. Finally, the test delivers clear end-point results which can help to optimize nutritional interventions in cattle.

## Additional files

Additional file 1: Table S1. Comparison of retinol concentrations in plasma (*n* = 132, obtained from Lab Germany) and serum (*n* = 61, obtained from Lab Japan) measured by HPLC and iCheck™ FLUORO (PDF 16 kb).

Additional file 2: Table S2. Concentrations of retinol in plasma (*n* = 10) measured by HPLC and iCheck™ FLUORO and whole blood measured by iCheck™ FLUORO (PDF 7 kb).

## Abbreviations

EDTA: Ethylenediaminetetraacetic acid
HPLC: High-performance liquid chromatography
iCheck™ FLUORO: Portable fluorometer for quantitative vitamin A determination
Lab DE: Laboratory located in Germany
Lab JP: Laboratory located in Japan
