# Supplementary material for: Validation of blood vitamin A concentrations in cattle: comparison of a new cow-side test (iCheck™ FLUORO) with high-performance liquid chromatography (HPLC)
Source: BMC Vet Res. 2017 May 10;13:126. doi: 10.1186/s12917-017-1042-3 (PMC5424361; doi:10.1186/s12917-017-1042-3)
Supplement: Supplementary file 1 — Comparison of retinol concentrations in plasma (n = 132, obtained from Lab Germany) and serum (n = 61, obtained from Lab Japan) measured by HPLC and iCheck™ FLUORO (PDF 16 kb). [file 12917_2017_1042_MOESM1_ESM.pdf]

**Table S1:** Retinol concentrations in plasma (Lab Germany; DE; $n$ = 132) and serum (Lab Japan; JP; $n$ = 61) measured by HPLC and iCheck$^{TM}$ FLUORO.

| Lab DE | | | Lab JP | | |
|---|---|---|---|---|---|
| **Sample #** | **HPLC** | **iCheck$^{TM}$** | **Sample #** | **HPLC** | **iCheck$^{TM}$** |
| | Retinol (mg/L) | Retinol (mg/L) | | Retinol (mg/L) | Retinol (mg/L) |
| | | | | | |
| 1 | 0.279 | 0.319 | 1 | 0.043 | 0.083 |
| 2 | 0.281 | 0.281 | 2 | 0.066 | 0.092 |
| 3 | 0.274 | 0.270 | 3 | 0.089 | 0.106 |
| 4 | 0.311 | 0.308 | 4 | 0.089 | 0.129 |
| 5 | 0.377 | 0.361 | 5 | 0.099 | 0.149 |
| 6 | 0.311 | 0.351 | 6 | 0.099 | 0.145 |
| 7 | 0.307 | 0.294 | 7 | 0.109 | 0.142 |
| 8 | 0.431 | 0.417 | 8 | 0.109 | 0.142 |
| 9 | 0.335 | 0.319 | 9 | 0.122 | 0.162 |
| 10 | 0.333 | 0.344 | 10 | 0.122 | 0.218 |
| 11 | 0.315 | 0.330 | 11 | 0.122 | 0.135 |
| 12 | 0.288 | 0.295 | 12 | 0.122 | 0.162 |
| 13 | 0.203 | 0.231 | 13 | 0.132 | 0.158 |
| 14 | 0.338 | 0.340 | 14 | 0.132 | 0.168 |
| 15 | 0.340 | 0.331 | 15 | 0.142 | 0.211 |
| 16 | 0.301 | 0.316 | 16 | 0.142 | 0.185 |
| 17 | 0.285 | 0.302 | 17 | 0.155 | 0.168 |
| 18 | 0.383 | 0.383 | 18 | 0.165 | 0.205 |
| 19 | 0.362 | 0.338 | 19 | 0.165 | 0.215 |
| 20 | 0.376 | 0.388 | 20 | 0.188 | 0.218 |
| 21 | 0.415 | 0.412 | 21 | 0.188 | 0.208 |
| 22 | 0.284 | 0.301 | 22 | 0.198 | 0.208 |
| 23 | 0.294 | 0.298 | 23 | 0.198 | 0.215 |
| 24 | 0.281 | 0.298 | 24 | 0.208 | 0.241 |
| 25 | 0.377 | 0.366 | 25 | 0.231 | 0.261 |
| 26 | 0.299 | 0.309 | 26 | 0.231 | 0.281 |
| 27 | 0.239 | 0.265 | 27 | 0.241 | 0.267 |
| 28 | 0.280 | 0.314 | 28 | 0.241 | 0.198 |
| 29 | 0.301 | 0.301 | 29 | 0.241 | 0.221 |
| 30 | 0.322 | 0.328 | 30 | 0.254 | 0.281 |
| 31 | 0.340 | 0.312 | 31 | 0.254 | 0.244 |
| 32 | 0.375 | 0.338 | 32 | 0.287 | 0.277 |
| 33 | 0.331 | 0.361 | 33 | 0.287 | 0.244 |
| 34 | 0.292 | 0.379 | 34 | 0.297 | 0.297 |
| 35 | 0.360 | 0.390 | 35 | 0.297 | 0.261 |
| 36 | 0.329 | 0.315 | 36 | 0.297 | 0.290 |
| 37 | 0.327 | 0.376 | 37 | 0.297 | 0.320 |
| 38 | 0.319 | 0.315 | 38 | 0.297 | 0.251 |
| 39 | 0.286 | 0.339 | 39 | 0.307 | 0.290 |

| | | | | | |
|---|---|---|---|---|---|
| 40 | 0.363 | 0.339 | 40 | 0.307 | 0.264 |
| 41 | 0.345 | 0.423 | 41 | 0.307 | 0.267 |
| 42 | 0.303 | 0.298 | 42 | 0.320 | 0.244 |
| 43 | 0.323 | 0.371 | 43 | 0.320 | 0.274 |
| 44 | 0.357 | 0.320 | 44 | 0.320 | 0.333 |
| 45 | 0.348 | 0.431 | 45 | 0.320 | 0.327 |
| 46 | 0.431 | 0.406 | 46 | 0.320 | 0.297 |
| 47 | 0.257 | 0.268 | 47 | 0.320 | 0.284 |
| 48 | 0.373 | 0.375 | 48 | 0.330 | 0.254 |
| 49 | 0.358 | 0.344 | 49 | 0.330 | 0.310 |
| 50 | 0.420 | 0.382 | 50 | 0.330 | 0.366 |
| 51 | 0.313 | 0.329 | 51 | 0.340 | 0.317 |
| 52 | 0.272 | 0.270 | 52 | 0.340 | 0.300 |
| 53 | 0.289 | 0.286 | 53 | 0.340 | 0.314 |
| 54 | 0.266 | 0.278 | 54 | 0.353 | 0.340 |
| 55 | 0.222 | 0.257 | 55 | 0.353 | 0.290 |
| 56 | 0.291 | 0.319 | 56 | 0.353 | 0.300 |
| 57 | 0.372 | 0.395 | 57 | 0.353 | 0.323 |
| 58 | 0.293 | 0.379 | 58 | 0.353 | 0.290 |
| 59 | 0.289 | 0.334 | 59 | 0.353 | 0.290 |
| 60 | 0.295 | 0.338 | 60 | 0.363 | 0.350 |
| 61 | 0.283 | 0.349 | 61 | 0.363 | 0.320 |
| 62 | 0.297 | 0.344 | | | |
| 63 | 0.240 | 0.290 | | | |
| 64 | 0.263 | 0.304 | | | |
| 65 | 0.298 | 0.338 | | | |
| 66 | 0.377 | 0.411 | | | |
| 67 | 0.246 | 0.285 | | | |
| 68 | 0.345 | 0.420 | | | |
| 69 | 0.193 | 0.232 | | | |
| 70 | 0.253 | 0.288 | | | |
| 71 | 0.304 | 0.372 | | | |
| 72 | 0.242 | 0.306 | | | |
| 73 | 0.300 | 0.348 | | | |
| 74 | 0.119 | 0.158 | | | |
| 75 | 0.086 | 0.107 | | | |
| 76 | 0.044 | 0.070 | | | |
| 77 | 0.137 | 0.172 | | | |
| 78 | 0.085 | 0.120 | | | |
| 79 | 0.053 | 0.082 | | | |
| 80 | 0.094 | 0.133 | | | |
| 81 | 0.061 | 0.091 | | | |
| 82 | 0.033 | 0.063 | | | |
| 83 | 0.135 | 0.166 | | | |
| 84 | 0.083 | 0.115 | | | |
| 85 | 0.044 | 0.080 | | | |
| 86 | 0.118 | 0.139 | | | |
| 87 | 0.075 | 0.102 | | | |
| 88 | 0.037 | 0.069 | | | |
| 89 | 0.198 | 0.232 | | | |

| | | | | | |
|---|---|---|---|---|---|
| 90 | 0.121 | 0.161 | | | |
| 91 | 0.200 | 0.223 | | | |
| 92 | 0.122 | 0.150 | | | |
| 93 | 0.217 | 0.233 | | | |
| 94 | 0.233 | 0.205 | | | |
| 95 | 0.355 | 0.320 | | | |
| 96 | 0.399 | 0.370 | | | |
| 97 | 0.409 | 0.350 | | | |
| 98 | 0.532 | 0.540 | | | |
| 99 | 0.315 | 0.290 | | | |
| 100 | 0.163 | 0.168 | | | |
| 101 | 0.413 | 0.438 | | | |
| 102 | 0.481 | 0.484 | | | |
| 103 | 0.257 | 0.259 | | | |
| 104 | 0.321 | 0.350 | | | |
| 105 | 0.377 | 0.311 | | | |
| 106 | 0.326 | 0.281 | | | |
| 107 | 0.253 | 0.227 | | | |
| 108 | 0.418 | 0.345 | | | |
| 109 | 0.367 | 0.298 | | | |
| 110 | 0.197 | 0.218 | | | |
| 111 | 0.400 | 0.365 | | | |
| 112 | 0.309 | 0.306 | | | |
| 113 | 0.236 | 0.264 | | | |
| 114 | 0.284 | 0.276 | | | |
| 115 | 0.463 | 0.406 | | | |
| 116 | 0.294 | 0.277 | | | |
| 117 | 0.425 | 0.354 | | | |
| 118 | 0.423 | 0.387 | | | |
| 119 | 0.384 | 0.340 | | | |
| 120 | 0.242 | 0.260 | | | |
| 121 | 0.176 | 0.176 | | | |
| 122 | 0.416 | 0.352 | | | |
| 123 | 0.323 | 0.294 | | | |
| 124 | 0.194 | 0.204 | | | |
| 125 | 0.308 | 0.282 | | | |
| 126 | 0.150 | 0.165 | | | |
| 127 | 0.394 | 0.313 | | | |
| 128 | 0.355 | 0.290 | | | |
| 129 | 0.227 | 0.232 | | | |
| 130 | 0.294 | 0.249 | | | |
| 131 | 0.261 | 0.237 | | | |
| 132 | 0.386 | 0.312 | | | |
