# Supplementary material for: Validation of blood vitamin A concentrations in cattle: comparison of a new cow-side test (iCheck™ FLUORO) with high-performance liquid chromatography (HPLC)
Source: BMC Vet Res. 2017 May 10;13:126. doi: 10.1186/s12917-017-1042-3 (PMC5424361; doi:10.1186/s12917-017-1042-3)
Supplement: Supplementary file 2 — Concentrations of retinol in plasma (n = 10) measured by HPLC and iCheck™ FLUORO and whole blood measured by iCheck™ FLUORO (PDF 7 kb). [file 12917_2017_1042_MOESM2_ESM.pdf]

Table S2: Concentrations of retinol in plasma (n = 10) measured by HPLC and iCheck^{TM} FLUORO and whole blood measured by iCheck^{TM} FLUORO.

**Sample #**	**HPLC**	**iCheck^{TM}**	**iCheck^{TM}**
	Plasma retinol (mg/L)	Plasma retinol (mg/L)	Blood retinol (mg/L)
1	0.231	0.244	0.245
2	0.310	0.327	0.299
3	0.422	0.404	0.375
4	0.310	0.300	0.344
5	0.416	0.377	0.371
6	0.521	0.451	0.480
7	0.305	0.304	0.307
8	0.312	0.294	0.294
9	0.398	0.394	0.369
10	0.309	0.307	0.322
